# Baryon correlations in Pythia

**DOI:** 10.1140/epjc/s10052-023-12271-7

**Published:** 2023-12-04

**Authors:** Leif Lönnblad, Harsh Shah

**Affiliations:** Department of Physics, Lund University, Sölvegatan 14A, 223 62 Lund, Sweden

## Abstract

We present the results from our investigation of angular correlations between baryons pairs in the Pythia8 event generator. We show how colour reconnection models and hadronization mechanisms influence such angular correlations and in particular address the effect of gluons on the baryon production mechanism in the Lund string fragmentation model. We conclude by discussing the new theoretical ideas in comparison with the ALICE $\textrm{pp}$ collision results for the baryon angular correlations. We propose a hypothesis for suppressing baryons produced in gluon jets and show how that may influence the angular correlations.

## Introduction

One of the research interests in particle physics is understanding the production mechanism and the spatial distribution of particles produced in high-energy particle collisions. This can be studied in various ways in colliders, e.g., by measuring single particle distributions as a function of one or more variables or looking at correlations between particles, for different species of particles. Using phenomenological models we can then use these measurements to gain theoretical insights into the underlying particle production mechanisms.

In this work, we address a long-standing open question about the angular correlations of pairs of the produced hadrons. A two-particle correlation function provides information regarding the production of another particle near the first particle. It is often studied as a function of relative pseudorapidity ($\Delta \eta$) and azimuthal angle ($\Delta \phi$) between two particles.

Depending on the chosen range of $\Delta \eta$, the angular distribution can be studied for long-range (large $\Delta \eta$) or short-range ($\Delta \eta \sim 0$). The long-range correlations around $\Delta \phi \sim 0$ are known as the near-side “ridge”. They are studied extensively in different collision systems like $\textrm{pp}$, $\textrm{p}A$, and $AA$ to understand the collective behaviour of the produced particles (see, e.g., [1] and references therein). The correlation function is defined such that it is unity for completely uncorrelated pairs of particles, and any correlation will show up as a larger value, while lower values indicate that there is an anti-correlation.

The short-range ($\mid \Delta \eta \mid < 1.3$) two-particle angular correlations were studied by the ALICE experiment in [2, 3] for low transverse momentum ($p_\perp < 2.5$ $\textrm{GeV}$) hadrons produced in $\textrm{pp}$ collisions at $\sqrt{s} =$ 7 $\textrm{GeV}$. This angular correlation study shows that the identified hadron pairs have different angular distributions depending on the types of hadrons in the pairs. The meson pairs of the same-sign and opposite-sign particles show correlations peak near $\Delta \phi = 0$ and a wide bump near $\Delta \phi = \pi$ (also known as the jet peak and the away-side ridge). On the other hand, baryons behave differently whether the angular distributions are produced for the same-sign or opposite-sign baryon pairs. For the opposite-sign baryon pairs the angular distribution is similar to that of the meson pairs, with a visible peak near $\Delta \phi = 0$ and almost flat distribution around $\Delta \phi = \pi$. For the same-sign baryon pairs, however, there is a clear anti-correlations near $\Delta \phi = 0$ (except for an indication of a tiny peak for $\Delta \phi =0$), and a broad peak is observed around $\Delta \phi = \pi$.

When comparing the ALICE experiment results with Pythia8 [4] generated events, the angular correlations for the same- and opposite-sign meson pairs are well reproduced, but Pythia is not able to reproduce the angular correlations for any of the baryon pairs types. It is also observed that this peculiar behaviour in the baryon sector is independent of the flavours of the baryons in the pairs, hence ruling out that the Fermi–Dirac correlation effects could play a major role. Some suggestions and hypothesises are proposed in [3]. Following these suggestions, recently Pythia ’s hadronization mechanism was studied by a theory group [5].

It can be noted that one of the heavy-ion collision experiments, STAR, measures anti-correlations around $\Delta \phi = 0$ for $\textrm{p}\bar{\textrm{p}}$ pairs produced in Au-Au collisions [6]. These results show that, unlike the observed correlations in $\textrm{pp}$ collisions, anti-correlations are observed for $\textrm{p}\bar{\textrm{p}}$ pairs in heavy-ion collisions. Furthermore, if we look into $\textrm{e}^+\textrm{e}^-$ collisions, then Pythia is able to reproduce the baryon angular correlations [7] in $\textrm{e}^+\textrm{e}^-$ collisions. These results from different collision systems reflect the non-triviality of the underlying physics of the angular correlations in the baryon sector. Hence we have decided to investigate the discrepancy in the angular correlations for baryons produced in $\textrm{e}^+\textrm{e}^-$ collisions and in $\textrm{pp}$ collisions. Moreover, we want to identify if any of the event simulation stages have any significant role in the baryon angular correlations.

Phenomenological models like Pythia play important roles in our attempts to quantify the initial and final state effects on the observables. Pythia is one of the successful general-purpose event generators, which can reproduce a variety of observables in good agreement with the data for $\textrm{e}^+\textrm{e}^-$ and $\textrm{pp}$ collisions for a wide range of collision energies. The partons are produced during the hard scattering, multiple partons interactions (MPIs) [8], and the parton showers, in stages in Pythia. These produced partons are then treated in terms of chains of colour dipoles between them, forming strings. An important feature in hadron collisions is that colour connections between the partons can be re-arranged by a colour reconnection (CR) [8, 9] model. After the CR, the colour singlet strings are hadronized by the Lund string fragmentation model [10] in Pythia. All these steps can influence the production rate of different hadrons, and correlations of the hadrons in the simulation results.

For simplicity, we keep our investigation limited to $\textrm{pp}$ collisions in this paper. We first have to understand which new effects appear in the event simulation when we move from $\textrm{e}^+\textrm{e}^-$ collisions to $\textrm{pp}$ collisions. Since Pythia8 is able to reproduce the angular correlations for the same- and opposite-sign meson pairs fairly well, we do not discuss the mesons’ angular correlations in the rest of the paper. Instead, our investigation is focused on the angular correlations of the same- and opposite-sign baryon pairs. We also keep our results limited to protons while discussing various theoretical aspects.

In the following, we will start in Sect. 2 by outlining the main baryon production mechanism in the Pythia8 implementation of the Lund string fragmentation model. Special attention is given to the role of gluons and how they may affect the production of baryons. This is followed by Sect. 3 where we discuss an alternative way of obtaining baryons in Pythia using the QCD-inspired colour reconnection model. In Sect. 4 we then discuss final-state effects and how they could affect baryon correlations, with special attention to the hadronic rescattering model. In Sect. 5 we then look at the phenomenology of these models and try to understand better what could cause the anti-correlation between like-sign baryons as found in data. Finally, in Sect. 6 we summarise with a discussion and an outlook.

**Fig. 1:**
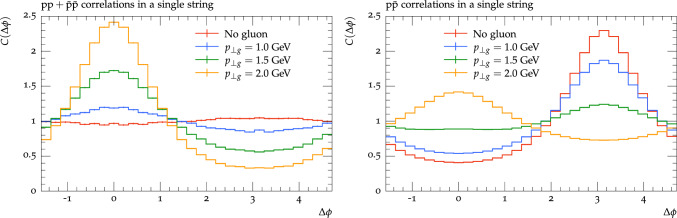
Azimuthal correlations along a string with or without a soft ($p_\perp =1.0$, 1.5, and 2.0 GeV) gluon. The left plot shows proton-proton ($\textrm{pp} +\bar{\textrm{p}}\bar{\textrm{p}}$) correlations, while the right shows proton–anti-proton correlations. The string is spanned between a quark and an anti-quark with opposite momenta ($p_{q/\bar{q}}=\pm 100$ GeV) along the *z*-axis and the gluons are placed at $\eta =0$. Only protons with $\mid \eta \mid <1$ are considered

## Baryons, popcorn and gluons in the Lund model

Throughout the perturbative phase of the generation of an event in Pythia, from multiple scatterings, initial- and final-state showers, the tracing of colour connections between partons is done using a leading-colour ($N_C\rightarrow \infty$) approximation. In hadronic collisions there is a possibility to rearrange these connections, as described below in Sect. 3, but the end results is in any case in colour-singlet *strings*, each connecting an anti-quark with a quark via a chain of colour-connected gluons. In the Lund string model, these strings are fragmented into hadrons as the string breaks by quark–anti-quark production in the string-like colour field between the partons.

The production rate of different hadron species depends on their quark content, mass, and spin. The quarks and anti-quarks of different flavours are produced in accordance with various parameters in the Lund String Fragmentation mechanism. The values of these parameters are primarily fixed from the model comparisons with LEP data. In a string breaking, a quark and an anti-quark are produced as virtual particles, which can come on-shell using the energy stored in the string through a tunnelling mechanism. Clearly, the production of heavier quarks would then need more of the string energy than light ones and are therefore suppressed.

The sequence of the further string break-ups will decide if the string piece will form a meson or a baryon as a primary hadron. A series of string breaks of multiple $q\bar{q}$ pairs will produce mesons. The simplest model for baryon production assumes that the string may break by the production of a diquark-antidiquark pair. This we call the ’diquark model’ in Pythia [11]. The consecutive string breaks of $q\bar{q}$ pairs on either side of the diquark and anti-diquark will form a baryon and an anti-baryon.

In the diquark model, the baryon and anti-baryon are always produced next to each other in rank, and therefore close in rapidity. Experimental results show that this is not the case always [12]. A mechanism was developed to add separation between a baryon and an anti-baryon produced next to each other in the same string. It is called a $\textit{popcorn mechanism}$ [13], and adds a possibility of meson production between the baryon and anti-baryon pair. The idea of the popcorn mechanism for baryon production is favoured by the experimental results [12]. At the moment, the popcorn mechanism is enabled by default although only one meson is allowed to form between the baryon and anti-baryon pair in Pythia8.

With or without popcorn, it is clear that we expect some correlations between baryons and anti-baryons. In particular, if we consider the case where they are produced next to each other along the string, their diquark and anti-diquark will have opposite transverse momentum along the string giving an anti-correlation in azimuth angle. However, there is no clear way of obtaining baryon-baryon correlation in the string fragmentation model as such. In a string, there must be at least one anti-baryon between two baryons, and the way transverse momentum is treated in the Lund model, there should be no correlation between them at all.

The MPI machinery for hadronic collisions produces many strings in an event, but they are hadronized independently and would not give rise to correlations between baryons from different strings. It has, however, been shown that the colour reconnection model in Pythia8 gives rise to radial flow [14], which in principle could be responsible for the correlations, and we will discuss that in Sect. 3. Irrespective of colour reconnection it is clear that the strings in hadronic collisions in general are connected to partons from MPI scatterings and are therefore not parallel to the beam axis.

In Fig. 1 we show how a jet peak evolves by comparing baryon azimuthal correlations in a single straight string, parallel to the beam axis, with the situation where this string has a (soft) gluon inserted, giving a transverse “kink”. For same-sign protons, the straight string has almost no correlations, but already a gluon with $p_\perp =1$ GeV will give a rather strong correlation. It can be noted that in Pythia8, around 80% of all hadrons in the central pseudo-rapidity bin come from string pieces connected to a parton with a $p_\perp$ of more than 1 GeV for a 7 TeV $\textrm{pp}$ collision. For $\textrm{p}\bar{\textrm{p}}$ we see, as expected, a strong anti-correlation since the di-quark breakup gives opposite transverse momenta for the baryon and anti-baryon. But we see that with a soft gluon, the anti-correlation reduced, and for a 2 GeV gluon it has been turned into a rather strong correlation.

In the MPI machinery, the string with a gluon would be accompanied by another string connected to a gluon going in the opposite azimuth direction. The latter would not be strongly correlated in rapidity, but would give rise to the so-called away-side ridge in a two-particle correlation spectra.

### Gluons vs. popcorn

Since we now have shown that gluon (mini-) jets contribute to the baryon angular correlations, it is relevant to scrutinise the baryon production in a Lund string with gluon “kinks” a bit closer.

**Fig. 2:**
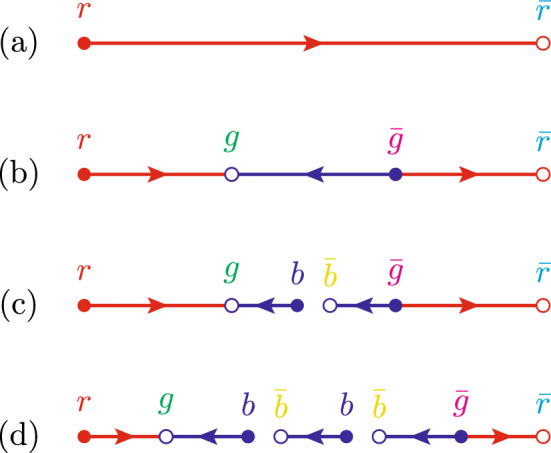
Illustration of the popcorn mechanism. In **a** no fluctuation has occurred, and a full string is spanned between a red – antired $q\bar{q}$ pair. In **b** a green – antigreen pair has appeared on the string as a quantum fluctuation. If the red and green quarks form an antiblue triplet, this reverses the colour flow in this part of the string, and the net force acting on the green quark is zero. In **c** the string breaks by the production of a blue – antiblue $q\bar{q}$ pair, resulting in two string pieces with diquark ends. In **d** another breakup in the blue triplet field results in an additional meson

The general idea behind the popcorn model used in Pythia is that the creation of a virtual $q\bar{q}$ pair in a string does not necessarily break the string. To do that it has to have the right colours such that the string is divided into two colour singlets. If the colour of the virtual pair does not match the colours of the string ends, the virtual fluctuation can then live for a while before the pair is annihilated again. As an example, in Fig. 2 we consider a string stretched between a red quark and an anti-red anti-quark, then imagine a virtual green–anti-green $q\bar{q}$ pair being created where the quark is moving towards the red end, and vice versa. The field between the virtual quarks will then effectively become antiblue–blue, and if another virtual pair occurs in this region the string can break. With the two quarks moving towards the quark end and two anti-quarks moving towards the anti-quark end. We created two string pieces, each carrying a non-zero baryon number.

For the $q\bar{q}$-fluctuation to live long enough for the string to break in between, the momenta of the *q* must be longitudinal towards the quark end of the string and vice versa for the $\bar{q}$. Any transverse momenta ($k_\perp$) would be suppressed with a factor $\propto \exp (-\pi k_\perp ^2/\kappa )$, where $\kappa$ is the string tension. Also, if the *q* and $\bar{q}$ had opposite momenta, the field in between would effectively be between two octet charges, which have more than twice the string tension^1^ giving rise to an extra attractive force between the virtual $q\bar{q}$ pair, making long-lived fluctuations heavily suppressed.

**Fig. 3:**
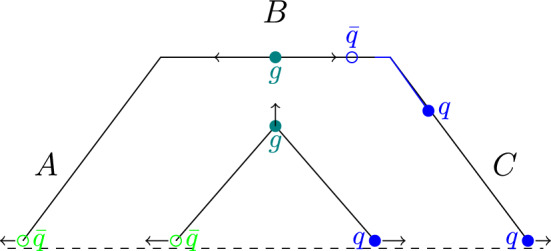
A schematic diagram shows two different phases of the movement of a $\bar{q}gq$ string, where the initial momentum of the *q*($\bar{q}$) is along the (negative) *z*-axis while the gluon momenta is perpendicular to them. The innermost lines represent the initial phase where a quark and an anti-quark are connected with a gluon kink in between. As the string stretches out and moves, the gluon gradually loses its energy to the string and eventually stops. At this point, the string cannot move further upwards, and the gluon kink is basically split into two kinks, and we enter the phase shown with the outermost lines. We thus end up with three pieces of straight string segments, *A*, *B*, and *C*. A virtual popcorn $q\bar{q}$ pair creation similar to Fig. 2b, but across a gluon kink between the string segments *B* and *C* is shown to highlight how the $q\bar{q}$ fluctuation has to propagate across a gluon kink with non-longitudinal momenta

This picture works nicely for a straight string. But if there is a gluon along a string, the picture changes. In Fig. 3 we show two snapshots of a string stretched between a quark and an anti-quark via a gluon. At first, there are two straight-string pieces (A and C) with the gluon as a kink. But the gluon is here retarded by two string pieces and will eventually stop, resulting in a new straight string piece (B) being formed, and the gluon kink is split into two. In Fig. 3 we show a virtual $q\bar{q}$ fluctuation across a gluon kink, and we can notice that *q* and $\bar{q}$ have non-longitudinal momenta components because they have to propagate to two different string segments (B and C).

In the current implementation of string fragmentation in Pythia8, there is no special treatment of baryon production close to such gluon kinks. From the description of the popcorn model above, however, it is clear that for a non-breaking virtual $q\bar{q}$ fluctuation, it would be very difficult for the quark or the anti-quark to propagate across such a kink. The pair should have only longitudinal momenta along the string piece where they are created, but the propagation across the kink corresponds to non-zero transverse momentum in the string piece on the other side of the kink, such fluctuations would be suppressed.

Since we have here shown that gluons are important for the azimuthal correlations between baryons we will in Sect. 5.3 use a toy model to investigate the possible effects of the suppression of baryon production close to gluon kinks.

## Junctions and colour reconnections

The popcorn and di-quark models are not the only way of obtaining baryons in Pythia. In some cases non-trivial colour topologies may arise prior to the string fragmentation stage, e.g., from the treatment of remnants in hadronic collisions, or when looking at baryon number violating BSM processes. In the MPI machinery, it is not uncommon that two (valence) quarks are taken from a proton, leaving a remnant in a colour-triplet state. Similarly, baryon number violating processes may decay a colour-triplet particle into two anti-triplet particles. In both cases, we may obtain colour-singlet string systems connecting three quarks (or three anti-quarks) in a so-called string junction topology [16]. Pythia8 is able to hadronise such systems, in a process that always will produce a net baryon number.

We will not be concerned with BSM here, and the junctions formed in the MPI remnant treatment mainly affects baryons in the far forward or backward regions of rapidity. There is, however, another way of creating junctions available in Pythia8, using the so-called QCD colour reconnection model [9].

CR models re-arrange the colour connections of the colour dipoles produced after MPIs and parton showers. The primary objective of CR is to reduce the net string length so that the model can reproduce the charged particles’ multiplicity and the observed enhancement in $\langle p_\perp \rangle$ $(N_{ch})$ distribution. Pythia has a default CR model, which is based on MPIs [8], where the different MPIs are colour reconnected in the $N_{c} \rightarrow \infty$ limit, and the only criteria to satisfy is to reduce the net string length.

**Fig. 4:**
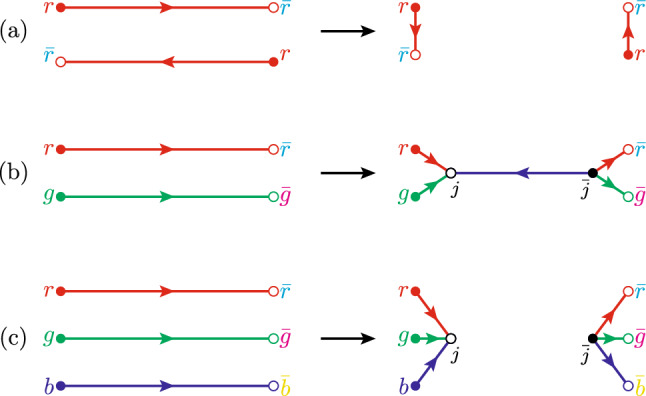
Illustration of the possible reconnections in the QCDCR model. **a** A “swing” between two dipoles in the same colour state. **b** Two dipoles in different colour states can form a connected junction–anti-junction system. **c** Three dipoles in different colour states form separate junction and anti-junction systems. In all cases, the total string length must be reduced in the process. Note that the dipole ends may be gluons that connect to other dipoles in a string system

Pythia8 has an alternative model, the QCDCR model [9], which follows QCD colour rules while performing CR. The QCDCR model allows the formation of junction systems, where two or three string pieces can be colour connected to a junction and an anti-junction system, each of which will produce at least one (anti-)baryon. The different colour reconnections possible in this model are summarised in Fig. 4. For case (a) two string pieces where the colour end of one is in a colour-singlet state w.r.t. the anti-colour end of the other, can reconnect in a so-called *swing*. In (b) we instead have the situation where the two (anti-) colour ends together are in an anti-triplet (triplet) state and can reconnect to two junction systems connected by a dipole. Finally, in (c) the (anti-) colour ends of three dipoles together form a colour singlet and can reconnect into a separate (anti-) junction system. In each case only reconnections that reduce the overall string lengths^2^ are allowed. This means that dipoles that are approximately anti-parallel in momentum space are more likely to reconnect like in case (a), while the opposite is true for cases (b) and (c).

The number of junctions in $\textrm{e}^+\textrm{e}^-$ collisions is very low, and it is pointed out in [9] that the effect of the QCDCR model is not clearly visible there. But in $\textrm{pp}$ collisions there are sometimes many MPIs, which enhance the possibility of junction formation during the CR. This means the QCDCR will produce additional baryons, on top of what is produced in the subsequent string fragmentation.

It should be noted that the two connected junctions can be separated by a long dipole or a chain of multiple dipoles that are reconnected and can separate the junctions by a large rapidity span. For example, the junction baryon and anti-baryon produced in Fig. 4 case (b) are often separated by multiple hadrons produced in between due to the fragmentation of the string piece connecting the two junctions. The junction systems in Fig. 4 case (c) are two independent colour singlet systems, and the baryon and the anti-baryon produced at junctions in these two systems are non-correlated. Hence the correlation between the resulting baryon and anti-baryon due to the junctions is much weaker than for the baryon–anti-baryon pairs produced in the string breaking. We can therefore expect that the correlations will be diluted by the additional baryons from the QCDCR model.

## Final-state effects on correlations

There are many potential final-state effects that may affect correlations between hadrons produced in the string fragmentation. The Lund group has studied several such models, e.g., a model for Fermi–Dirac correlations [17], the so-called rope hadronisation model [15] and a model for repulsion between strings [18]. Of these, the rope model mainly affects the flavour composition, and is not expected to give significant effects on correlations. Also, the string repulsion will give a flow effect in high multiplicity $\textrm{pp}$ events, but the effect is overall quite small in $\textrm{pp}$, and it will increase correlations both at $\Delta \phi =0$ and $\Delta \phi =\pi$ and would therefore not improve the description of baryon-baryon correlations in Pythia8 at small angles. Fermi–Dirac effects would decrease the correlation at small angles for identical baryons, but again the effect is expected to be small.^3^ Also, as already pointed out in [5], the effect found in [2, 3] is the same for $\textrm{pp}$ and $\textrm{p}\Lambda$, this can also not improve the situation.

Instead, we will focus on the model for hadronic rescattering [19, 20]. By following the production vertices of all partons in the event, it is possible to calculate the production points of all hadrons in the string fragmentation [21]. Then one can study the possible scatterings between these hadrons in a way similar to the UrQMD [22] and SMASH [23] models. Clearly, the rescatterings will mainly affect hadrons that are propagating in the same general direction, and one may expect that it will reduce correlations at $\Delta \phi =0$, and we will therefor investigate this model in the following section.

## Comparison with data

So far we have presented a set of ideas that may affect baryon correlations in Pythia, and in this section, we will confront these ideas with data. It can be noted that we have also tested varying standard string fragmentation parameters, such as flavour ratios, di-quark production rate, spin ratios of the di-quarks, and $p_\perp$ assignment to the produced hadrons. We found, however, that none of these changes significantly affects the angular correlations of the same-sign baryon pairs in Pythia.

In Ref. [5], the main conclusion was that only by forcing Pythia8 to produce at most one di-quark breakup per string, it was possible to understand the correlations found in data. Such an artificial change in the behaviour of the string fragmentation is of course not a satisfactory solution, but it gives us hints as to what is needed. We will therefore concentrate on *reducing* the number of di-quark breakups in strings with (semi-) hard gluons, but also to introduce alternative baryon production mechanisms that do not exhibit the correlations found in string fragmentation. In addition, we will also consider final state effects from hadronic rescattering.

In all simulations, we have used the Pythia version 8.306 to generate $\textrm{pp}$ events at $\sqrt{s}=7$ TeV. The analysis of the generated events was done using the Rivet [24] routine ALICE_2016_I1507157 which mimics the analysis in [3].^4^

### The QCD colour reconnection model

**Fig. 5:**
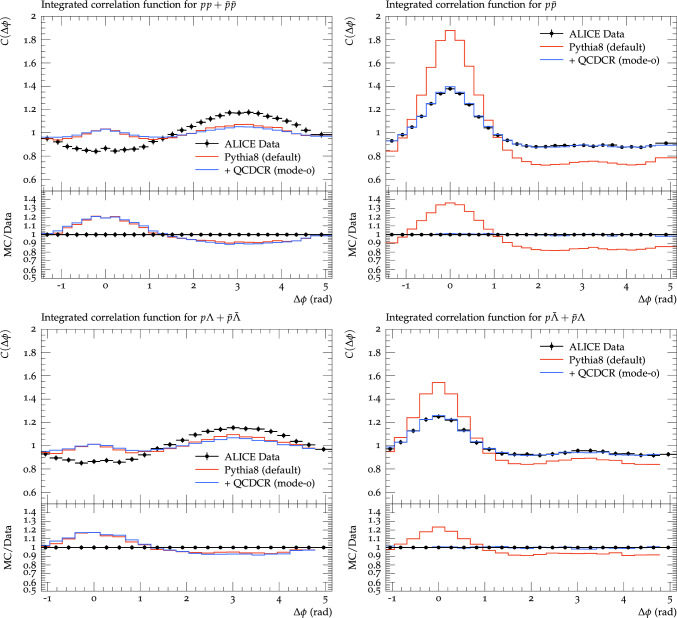
Baryon azimuthal correlations. *Top*: $\textrm{pp}$ + $\bar{\textrm{p}}\bar{\textrm{p}}$ pairs on the left, and $\textrm{p}\bar{\textrm{p}}$ pairs on the right. *Bottom*: $\textrm{p}\Lambda$ + $\bar{\textrm{p}}\bar{\Lambda }$ pairs on the left, and $\textrm{p}\bar{\Lambda }$ + $\bar{\textrm{p}}\Lambda$ pairs on the right. Events are generated for $\textrm{pp}$ collisions at $\sqrt{s}=7$ TeV and are compared with ALICE data [3]. The red and blue lines represent results using the Pythia8 Monash tune with MPIs-based colour reconnection, and using QCDCR (mode-0) colour reconnection respectively

We begin with the QCDCR model, which introduces a completely new way of producing baryons. We have used the so-called “mode-0” tune presented in [9], with no further changes. The results are presented in Fig. 5 and show a remarkable improvement in the baryon–anti-baryon correlations as compared to the default CR model in Pythia8.

The choice of the CR model does not, however, improve the angular (anti-) correlations for the same-sign baryon pairs. The effect is rather a general reduction in correlations, which is expected since the model will produce additional baryons with fewer correlations.

The reduction is more clearly seen for the opposite-sign baryon pairs, where QCDCR model reduces the amplitude of the baryon-antibaryon pair correlations near $\Delta \phi =0$, and also reduces the corresponding away side anti-correlation, bringing the simulation results in agreement with the data. It is clear that the separation between the junction and anti-junction systems created by the QCDCR model plays a significant role in improving the angular correlations between the opposite-sign baryon pairs.

It should be noted that we have also studied the effects in meson correlations (which are not shown here) but found no significant effect of the choice of CR model there.

Since the QCDCR shows significant improvement in the angular correlations of the opposite-sign baryon pairs, we will in the following use the QCDCR as our base-line set-up when adding other modifications.

### Hadronic rescattering

**Fig. 6:**
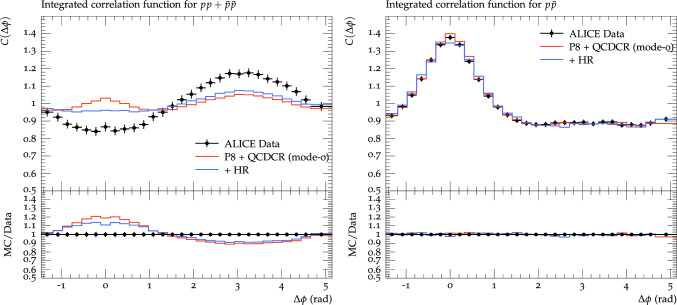
Proton azimuthal correlations for $\textrm{pp}$ + $\bar{\textrm{p}}\bar{\textrm{p}}$ pairs on the left, and $\textrm{p}\bar{\textrm{p}}$ pairs on the right. Events are generated for $\textrm{pp}$ collisions at $\sqrt{s} = 7$ TeV and are compared with ALICE data [3]. The red lines show results from Pythia8 with QCDCR (mode 0), while blue lines show the same but with hadronic rescattering

The produced hadrons can interact with nearby hadrons via elastic or inelastic scattering. A model for hadronic rescattering [19] was recently added in Pythia, implementing $2 \rightarrow 2$ and $2 \rightarrow 3$ type inelastic and elastic hadronic rescatterings.^5^ The naive expectation is that rescattering will blur preexistent correlations between particles going in the same direction, and that is indeed what is seen for the $\textrm{p}\bar{\textrm{p}}$ correlations in Fig. 6. The effect is not very large, but we know that rescattering effects in general are quite modest in $\textrm{pp}$ collisions and stronger only in high multiplicity events. We note, however, that for the like-sign proton correlations in Fig. 6 the effect is much more visible. In fact, the correlation around $\Delta \phi =0$ is all but wiped out.

The reason for this is somewhat non-trivial, and is related to the annihilation of baryons–anti-baryon pairs in the rescattering. As explained in Sect. 2, the peak at $\Delta \phi =\Delta \eta =0$ mainly comes from jets, where the two particles are typically produced in the same string. For $\textrm{p}\bar{\textrm{p}}$ pairs the main contribution is pairs produced in a single diquark breakup, and since the diquark and anti-diquark will have opposite transverse momenta along the string, it is very unlikely that the baryons formed would rescatter with each other. For $\textrm{pp}$ and $\bar{\textrm{p}}\bar{\textrm{p}}$ pairs, however, we would need two baryon–antibaryon pairs produced close together along the string and a baryon in one pair could then more easily annihilate with the anti-baryon in the other. This effect turns out to be rather large. Adding rescattering to the $p_{\perp g}=2$ GeV runs in Fig. 1 does not affect the shape of the correlations very much, but the number of like-sign pairs will be reduced by around 40%. We, therefore, conclude that the reason for the relatively large effect for $\textrm{pp}$ and $\bar{\textrm{p}}\bar{\textrm{p}}$ in Fig. 6 is that the number of pairs stemming from the same string is reduced.

### Suppressing baryon production close to gluon kinks

**Fig. 7:**
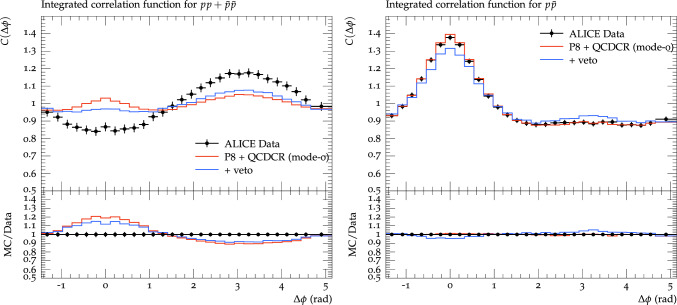
Proton azimuthal correlations for $\textrm{pp}$ + $\bar{\textrm{p}}\bar{\textrm{p}}$ pairs on the left, and $\textrm{p}\bar{\textrm{p}}$ pairs on the right. Events are generated for $\textrm{pp}$ collisions at $\sqrt{s} = 7$ TeV and are compared with ALICE data [3]. The red lines show results from Pythia8 with QCDCR (mode-0), while blue lines show the same but with a veto on primary baryons spanning a gluon kink as explained in the text

**Fig. 8:**
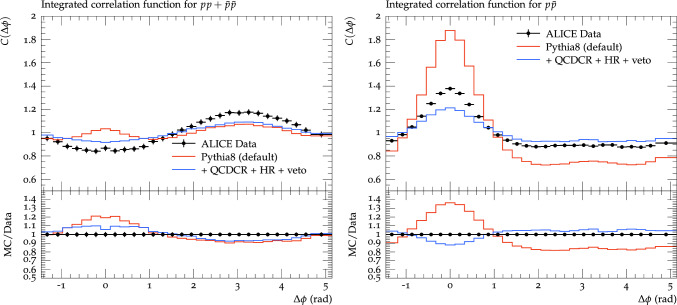
Proton azimuthal correlations for $\textrm{pp}$ + $\bar{\textrm{p}}\bar{\textrm{p}}$ pairs on the left, and $\textrm{p}\bar{\textrm{p}}$ pairs on the right. Events are generated for $\textrm{pp}$ collisions at $\sqrt{s} = 7$ TeV and are compared with ALICE data [3]. The red lines show results from the default Pythia8, while blue lines show the result with QCDCR (mode-0) colour reconnection and hadronic rescattering (HR) switched on and with a veto on primary baryons spanning a gluon kink as explained in the text

There is currently no proper implementation for the possible suppression of baryon production in string fragmentation close to gluon kinks in the popcorn mechanism discussed in Sect. 2.1. Instead, we will study a simplified toy model to understand what the effects may be.

We have decided to constrain the baryon production using the $\texttt {UserHooks}$ facility in Pythia8 [4], which allows a user to intervene at different stages of the event generation. In particular, there are options to intervene in the string fragmentation procedure and one possibility is to simply veto the production of a single hadron, based on additional criteria implemented by the user.

In our crude implementation, we veto any baryon produced in a diquark breakup if the previous breakup was in a different string region. As an example consider the case in Fig. 3 where the gluon has lost all its energy. If there has been a normal $q\bar{q}$ breakup in string region **C** and the next breakup is a diquark–anti-diquark breakup in region **B**, we veto the baryon to be produced, and tell Pythia to try another breakup instead. It should be noted that the Lund string fragmentation model is left–right symmetric, and if we go from the other ($\bar{q}$) end and the same diquark breakup occurred in region **B** the following $q\bar{q}$ breakup in **C** producing the same baryon from a *kinky* string piece, is not vetoed. The reason for implementing it in this way is technical, but effectively it will result in a suppression of baryons produced around a string corner with a factor of 0.5.

In Fig. 7 see the effect of applying this toy model to Pythia8 including QCDCR. As expected the jet peak for baryon pairs is reduced, and for $\textrm{pp} +\bar{\textrm{p}}\bar{\textrm{p}}$ the lines are moved closer to the data. Unfortunately, the reduction of the jet peak is also present for $\textrm{p}\bar{\textrm{p}}$, which worsens somewhat the excellent agreement with data obtained from the QCDCR model.

It should be noted that our toy model will reduce the overall number of baryons produced in general, even in $\textrm{e}^+\textrm{e}^-$ collisions, since also there we have gluon kinks. To be completely fair we should therefore have retuned the parameters affecting baryon production to obtain the same reproduction of LEP data. We would, however, expect the reduction of the jet peak to stay more or less the same.

As a final result, we show in Fig. 8 a comparison between the default Pythia and the accumulated changes of all three models investigated her: QCDCR, hadronic rescattering and the vetoing of baryons close to gluon kinks. We see that the reproduction of the ALICE date is far from perfect if the models are added, but there is a clear improvement over the default Pythia8. The jet peak is reduced both for $\textrm{pp}$ +$\bar{\textrm{p}}\bar{\textrm{p}}$ and $\textrm{p}\bar{\textrm{p}}$, and we see that there is even an anti-correlation for like-sign proton pairs around $\Delta \phi =\Delta \eta =0$.

## Discussion and summary

We have shown that the observed anti-correlation for the same-sign baryon pairs in the ALICE experiment for $\textrm{pp}$ collisions is a non-trivial outcome of many-fold effects. We also presented the first steps towards understanding the failure of Pythia in reproducing these experimental results. We found that two already existing models in Pythia8, the QCDCR model and the hadronic rescattering model, have a significant effect on the correlations, and adding these to the default Pythia8 improves the description of data significantly.

The QCDCR model produces additional baryons due to junction systems forming as a part of the reconnections of the colour dipoles. Such junction baryons are much less correlated than those produced in string fragmentation. The string connecting two junctions can produce multiple hadrons between the two junction baryons, unlike the popcorn baryons, which are separated by only one meson in between. We show that it visibly reduces the correlations between the opposite-sign baryon pairs in the jet peak near $\Delta \phi =0$. As a result, Pythia is able to reproduce the angular correlation distribution for the opposite-sign baryon pairs.

The anti-correlations in the same-sign baryon pairs are rather complex results. Although the QCDCR model improves the Pythia results, it is not sufficient. Adding the hadronic scattering model, we found that the effect of annihilation of baryon–anti-baryon in jets with more than one baryon–anti-baryon pair is quite significant, while if there is only one pair, there is typically no annihilation. This gives a further reduction of the jet peak for same-sign baryons, while the effects on unlike-sign correlations are small. Still, the jet peak for same-sign baryons in Pythia needs to be further reduced in order to reproduce data.

The authors in [5] managed to make Pythia reproduce data, by forcibly forbidding more than one baryon–anti-baryon pair to be produced in a string. This effectively removed the jet peak in the same-sign baryon correlations, leaving only the anti-correlation in the away-side ridge. Here we instead propose a more physical mechanism, where baryon production close to gluon kinks in a string is suppressed. The motivation for this comes from the popcorn model of baryon production in a string. Here an extra non-breaking virtual $q\bar{q}$ is required to exist before a $q\bar{q}$ pair breaking occurs to produce an effective di-quark breakup, and we argue that it is less likely to have such an extra pair close to a gluon kink.

Since the jet peak around $\Delta \phi =\Delta \eta =0$ in the angular correlations in $\textrm{pp}$ collisions mainly consist of particle pairs from the same (mini-) jet (which at the LHC is likely to be a gluon jet), one would then expect a reduction of the peak for baryon pairs in general. For same-sign baryon pairs, we expect the reduction to be even bigger since we require two such popcorn breakups in the same string. We have here qualitatively confirmed that this is the case using a toy model, where we simply disallow some such breakups close to gluon kinks, which has motivated us to attempt a more realistic modelling of the effect in the future. Such a model would have to take into account the size of the transverse momentum of the gluon kink, as well as the distance between the breakup and the kink. Since the overall number of baryons would be reduced, such a model would also require a proper retuning of the baryon parameters in Pythia8, but it is still likely that the jet peak for same-sign baryon correlations would be reduced. Whether it will be reduced enough to reproduce data remains to be seen.

We believe that the baryon suppression near gluon kinks will affect the baryon-to-meson ratios in $\textrm{e}^+\textrm{e}^-$ collisions as well. Hence the model should be retuned to LEP data for $\textrm{e}^+\textrm{e}^-$ collisions in future.

Finally, we note that there are other independent measurements that could verify our hypothesis of suppressed baryon production close to gluons. One obvious example is to compare the baryon-to-meson ratio inside a gluon jet to that of a quark jet, which could be done by comparing inclusive jets to jets produced together with hard photons. We are not aware of any study where the jet substructure has been studied for identified hadrons, but we would certainly like encourage our experimental colleagues to pursue such measurements at the LHC.

## Data Availability

This manuscript has no associated data or the data will not be deposited. [Authors’ comment: The work done in this paper is a phenomenological modifications in the Pythia model. The experimental data used in this work is cited appropriately. Hence, there is no data to be deposited with this paper.]

## Footnotes

1 See, e.g., [15].

2 The *length* of a string is approximately given by the sum of the logarithm of the invariant masses of the dipoles forming the string (see Ref. [9] for details).

3 We have confirmed that the effect is small by making a rudimentary implementation in Pythia8 of the Fermi–Dirac model described in [17].

4 Note that we have made slight corrections to the kinematical cuts used for different particle species in the Rivet routine, to better reflect the cuts made in the experiment. These corrections will be included in a future release of Rivet.

5 It should be noted that the absence of $3 \rightarrow 2$ scatterings means that the overall multiplicity of an event is slightly increased. In [19] this was compensated by a slight change in the $p^{Ref}_{T0}$ parameter regulating the divergences in the MPI scattering cross sections. We have checked that a similar increase does not significantly influence the correlation results presented here.

## References

[1] Khachatryan V (2010). Observation of long-range near-side angular correlations in proton-proton collisions at the LHC. JHEP.

[2] Graczykowski LK, Janik MA (2014). Angular correlations measured in pp collisions by ALICE at the LHC. Nucl. Phys. A.

[3] Adam J (2017). Insight into particle production mechanisms via angular correlations of identified particles in pp collisions at $$\sqrt{\rm s}=7$$ TeV. Eur. Phys. J. C.

[4] Bierlich C  (2022). A comprehensive guide to the physics and usage of PYTHIA 8.3. SciPost Phys. CodeBases.

[5] Demazure N, Sebastián VG, Llanes-Estrada FJ (2023). Baryon anticorrelations in PYTHIA. Few Body Syst..

[6] Adam J (2020). Beam-energy dependence of identified two-particle angular correlations in $$\sqrt{s_{NN}}$$ = 7.7–200 GeV Au+Au collisions. Phys. Rev. C.

[7] Aihara H (1986). Study of baryon correlations in $$e^+e^-$$ annihilation at 29-GeV. Phys. Rev. Lett..

[8] Sjöstrand T, van Zijl M (1987). A multiple interaction model for the event structure in Hadron collisions. Phys. Rev. D.

[9] Christiansen JR, Skands PZ (2015). String formation beyond leading colour. JHEP.

[10] Andersson B, Gustafson G, Ingelman G, Sjöstrand T (1983). Parton fragmentation and string dynamics. Phys. Rep..

[11] Andersson B, Gustafson G, Ingelman G, Sjöstrand T (1982). Baryon production in lepton–nucleon scattering and diquark fragmentation. Z. Phys. C.

[12] Aihara H (1985). Baryon production in $$e^+e^-$$ annihilation at $$\sqrt{s}=29$$ GeV: clusters, diquarks, popcorn?. Phys. Rev. Lett..

[13] Andersson B, Gustafson G, Sjöstrand T (1985). Baryon production in jet fragmentation and $$\Upsilon $$ decay. Phys. Scripta.

[14] Ortiz Velasquez A , Christiansen P, Cuautle Flores E, Maldonado Cervantes I, Paić G (2013). Color reconnection and flowlike patterns in $$pp$$ collisions. Phys. Rev. Lett..

[15] Bierlich C , Gustafson G, Lönnblad L, Tarasov A (2015). Effects of overlapping strings in pp collisions. JHEP.

[16] Sjöstrand T, Skands PZ (2003). Baryon number violation and string topologies. Nucl. Phys. B.

[17] Duran Delgado RM, Gustafson G, Lönnblad L (2007). String effects on Fermi–Dirac correlation measurements. Eur. Phys. J. C.

[18] C. Bierlich, G. Gustafson, L. Lönnblad, A shoving model for collectivity in hadronic collisions (2016). arXiv:1612.05132 [hep-ph]

[19] T. Sjöstrand, M. Utheim, A framework for Hadronic rescattering in pp collisions. Eur. Phys. J. C **80**(10), 907 (2020). 10.1140/epjc/s10052-020-8399-3. arXiv:2005.05658 [hep-ph]

[20] Bierlich C, Sjöstrand T, Utheim M (2021). Hadronic rescattering in pA and AA collisions. Eur. Phys. J. A.

[21] Ferreres-Solé S, Sjöstrand T (2018). The space-time structure of hadronization in the Lund model. Eur. Phys. J. C.

[22] Bass SA (1998). Microscopic models for ultrarelativistic heavy ion collisions. Prog. Part. Nucl. Phys..

[23] Weil J (2016). Particle production and equilibrium properties within a new hadron transport approach for heavy-ion collisions. Phys. Rev. C.

[24] Bierlich C (2020). Robust independent validation of experiment and theory: Rivet version 3. SciPost Phys..

